# Ultra-high-resolution subtraction CT angiography in the follow-up of treated intracranial aneurysms

**DOI:** 10.1186/s13244-019-0685-y

**Published:** 2019-01-28

**Authors:** Frederick J. A. Meijer, Joanne D. Schuijf, Joost de Vries, Hieronymus D. Boogaarts, Willem-Jan van der Woude, Mathias Prokop

**Affiliations:** 1 Department of Radiology and Nuclear Medicine, Radboud University Medical Center, Geert Grooteplein 10, P/O Box 9101, 6500 HB Nijmegen, The Netherlands; 2 Canon Medical Systems Europe B.V., Global Research & Development Center, Zoetermeer, The Netherlands; 3 Department of Neurosurgery, Radboud University Medical Center, Nijmegen, The Netherlands

**Keywords:** Multi-detector row computed tomography, Angiography, Brain, Cerebral aneurysm

## Abstract

In subtraction CT angiography (CTA), a non-contrast CT acquisition is subtracted from a contrast-enhanced CTA acquisition. Subtraction CTA can be applied in the detection, classification, and follow-up of intracranial aneurysms and is advantageous over conventional angiography because of its non-invasive nature, shorter examination time, and lower costs. Recently, an ultra-high-resolution CT scanner has been introduced in clinical practice offering an in-plane spatial resolution of up to 0.234 mm, approaching the resolution as seen during conventional invasive digital subtraction angiography (DSA). The twofold increase in spatial resolution as compared to a conventional CT scanner could improve the evaluation of small vascular structures and, coupled with dedicated post-processing techniques, further reduce metal artifacts. Technical considerations using a state-of-the-art high-resolution subtraction CTA protocol are discussed for application in the follow-up of surgical and endovascular treated intracranial aneurysms.

## Key points

- Head subtraction CT angiography can be applied in the follow-up of treated cerebral aneurysms and could obviate conventional angiography.
- Ultra-high-resolution subtraction CT angiography is feasible and provides superior image quality in comparison to standard resolution subtraction CT angiography, at similar radiation dose.
- The added value of ultra-high-resolution CT angiography still needs to be evaluated in future prospective cohort studies.

## Introduction

In subtraction CT angiography (sCTA), a non-contrast CT acquisition is subtracted from a contrast-enhanced CTA acquisition. sCTA has shown to produce comparable results to digital subtraction angiography (DSA) in the detection and classification of intracranial aneurysms [1–3]. sCTA is advantageous over DSA because of its non-invasive nature, shorter examination time, and lower costs. Small aneurysms and aneurysms adjacent to bony structures are more accurately detected in sCTA as compared to conventional CTA [1–3]. Furthermore, sCTA can be applied in the follow-up of treated aneurysms after surgical clip placement, endovascular stent, or coil occlusion [4]. In order to reduce metal artifacts, metal artifact reduction (MAR) algorithms like single-energy metal artifact reduction (SEMAR) can be applied [5]. sCTA with conventional CT systems can achieve results comparable to conventional DSA in the follow-up of flow diverter- and surgical clip-treated patients [4, 6]. However, DSA is still commonly applied in clinical practice because of its high spatial resolution and the ability of selective vessel catheterization.

Recently, an ultra-high-resolution CT (UHRCT) scanner has been introduced in clinical practice [7]. This system provides a more than twofold increase in spatial resolution as compared to a conventional CT scanner. Coupled with dedicated post-processing techniques, this system allows non-invasive vascular imaging with fine detail, approaching the resolution as seen during invasive DSA. The increase in spatial resolution enables improved depiction of small aneurismal remnants and small caliber vessels. Accordingly, UHR sCTA may further extend the applicability of CT in the follow-up of patients with treated intracranial aneurysms. However, at this moment, experience with this novel system remains limited.

In this manuscript, we discuss technical considerations using a state-of-the-art UHR sCTA protocol for clinical application in the follow-up of treated intracranial aneurysms. Technical features of the UHRCT system and available post-processing algorithms are described followed by clinical examples illustrating our initial clinical experience with this novel system.

## Technical considerations

### Ultra-high-resolution CT (UHRCT)

Recently, an UHRCT scanner (Aquilion Precision, Canon Medical Systems, Otawara, Japan) has been introduced, offering an in-plane spatial resolution of up to 0.234 mm (1024 × 1024 matrix) [7]. This UHRCT system is a 160-row multi-detector row system with superfine detector grids providing an effective detector element size of 0.25 mm × 0.25 mm, which is half the detector element size of a conventional high-end CT system. Likewise, the system has 1792 channels as opposed to the 896 channels seen in conventional CT scanners. Ultra-thin interseptal gaps between the detector elements maximize the light-sensitive areas on the detector. Combined with a data acquisition system redesigned for UHRCT, the resulting image noise remains comparable to conventional CT. The CT system features an adaptive focal spot X-ray tube with a minimum focus size as small as 0.4 mm × 0.5 mm. Gantry rotation time of 0.35 s can be achieved. In addition to the conventional 512 × 512 matrix, reconstructions are also possible with 1024 × 1024 and 2048 × 2048 matrix sizes in order to reduce pixel size.

### Image reconstruction algorithms

To reduce the patient radiation dose, new image reconstruction techniques are continuously being developed for noise reduction and image quality improvement. Iterative reconstruction (IR) techniques are therefore rapidly replacing the traditional filtered back projection (FBP) reconstructions. On UHRCT, hybrid iterative reconstruction algorithms as well as the more recent full model-based iterative reconstruction (MBIR) are available [8]. All are optimized for UHRCT acquisitions allowing noise reduction while preserving spatial resolution and structural edges. The MBIR algorithm (FIRST, Forward projected model-based Iterative Reconstruction SoluTion) is based on a forward projection model which accurately models system geometry, optics, and cone angle and a statistical model that models the noise characteristics in the measurements. For each iteration, the image is forward projected to the projection space and then compared to the original projection data. In addition, a regularization function optimized for the anatomical region is applied. An updated image is calculated by evaluating the mismatch between the forward-projected data and original projection data. The updated images are then combined to produce a new image which is a refinement of the previous image with improved spatial resolution and reduced noise. This process repeats itself until a final optimal image is obtained. The iterative reconstruction techniques are integrated into the automatic exposure settings for optimal dose reduction.

### Metal artifact reduction (MAR)

On CT, metal causes beam hardening artifacts and scattering that degrade image quality and lead to decreased diagnostic accuracy for evaluating adjacent structures. To reduce these artifacts, software-based MAR, such as single-energy metal artifact reduction (SEMAR), can be applied on both single- and dual-energy acquisitions and does not require a dedicated system or acquisition technique [5]. Briefly, the process starts with automatic segmentation of metal parts in an original first-pass image. Then, forward projection is applied to find the metal trace in the sinogram. The metal trace is removed through linear interpolation in the sinogram using nearby non-metal measurements, and an interpolated sinogram is reconstructed to create a second-pass image. This interpolation-corrected image is then segmented into different tissue classes (air, water, bone). The combined image is forward reprojected onto the metal trace using linear integration of interpolated voxels. Using a linear baseline shift approach, the original sinogram is blended with the forward-reprojected tissue-classified image on the metal trace. The blended sinogram is reconstructed to create a third-pass image, and the metal image (without artifacts) is added to create the final image.

### Subtraction CTA

Subtraction is a post-processing technique to eliminate high-density structures, such as bone, from CT images. A subtraction dataset is obtained by subtracting a non-contrast acquisition (mask image) from a contrast-enhanced image. For this, accurate registration of the two datasets is essential. In contrast to DSA, subtraction CT is 3D based and therefore more challenging. In addition, the (minimal) time delay between the non-contrast and contrast acquisitions can introduce differences between scans due to patient motion or vascular pulsation. To overcome these challenges, a dedicated registration algorithm is applied. This deformable registration algorithm matches the position of bony structures and calcifications on the mask image to the contrast scan prior to subtraction. Accordingly, subtracted image data are obtained from which high-density structures have been removed and which can be used for evaluation in conjunction with the conventional contrast-enhanced images.

### UHR cerebral subtraction CTA protocol

**Fig. 1:**
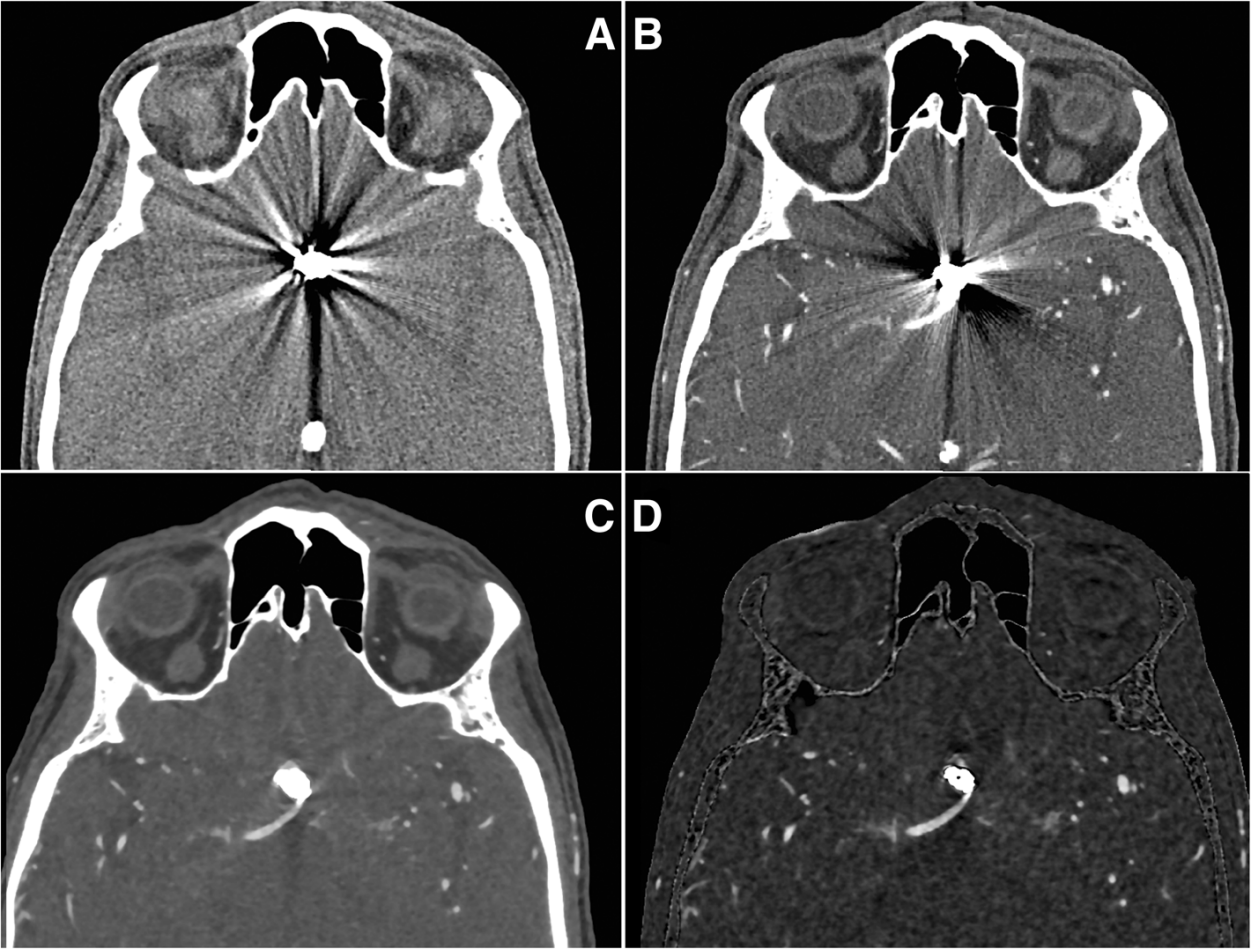
Impact of post-processing on image quality and metal artifact reduction. **a** Non-enhanced CT and **b** CT angiography with pronounced scattering artifacts of a coil-treated anterior communicating artery aneurysm. **c** CT angiography with metal artifact reduction (MAR) and model-based iterative reconstruction (MBIR) providing substantial artifact reduction. **d** Subtraction CT angiography with MAR and MBIR filtering providing further artifact reduction and resulting in adequate image quality for diagnostic evaluation

Image acquisition with UHRCT is comparable to the standard 80-row CT scanning. A typical protocol for cerebral sCTA with UHRCT consists of two consecutive high-resolution scans, one pre-contrast and one contrast-enhanced, using orbital synchronization. Orbital synchronization allows consecutive image acquisition along the same helical path, thus improving the quality of registration of two datasets and, consequently, improving subtraction accuracy. Scanning is performed in super high-resolution mode, without ECG gating, using the following parameters: collimation 0.25 mm, tube voltage 100 kV, tube current determined via automatic exposure control, focus size down to 0.4 × 0.5 mm, pitch factor 0.8, and a gantry rotation time of 1.0 s. A bolus of 50-ml contrast medium is injected with an injection rate of 5 ml/s followed by a saline flush. Following acquisition, images are automatically reconstructed with a 0.25-mm thickness, a 0.25-mm interval, and a 240-mm FOV, using MBIR (FIRST Brain CTA) integrated with MAR (SEMAR). The pre- and post-contrast scans are automatically registered and subtracted, providing a subtraction CT dataset in addition to the standard reconstructions. An example illustrating the impact of the available post-processing techniques (combined MAR and MBIR followed by subtraction) on metal artifact reduction and image quality in a patient with aneurismal coil embolization is provided in Fig. 1.

## Clinical application

Patients with a treated intracranial aneurysm need a follow-up to evaluate the level of aneurysm occlusion, or possible recanalization [9]. Both DSA and sCTA can be considered in the follow-up of a surgical clip or endovascular stent-treated cerebral aneurysm, whereas MRA is not reliable in these patients due to susceptibility artifacts. In our hospital, we routinely perform sCTA for this indication, and DSA is performed only in case the image quality of sCTA is suboptimal or when there is doubt about the aneurysm occlusion. Although MRA is the preferred imaging modality for the follow-up of endovascular coil-treated aneurysms, some patients cannot undergo an MRI examination due to unsafe implants (e.g., pacemaker) or because of claustrophobia. In these cases, sCTA can be considered as an alternative to DSA.

**Fig. 2:**
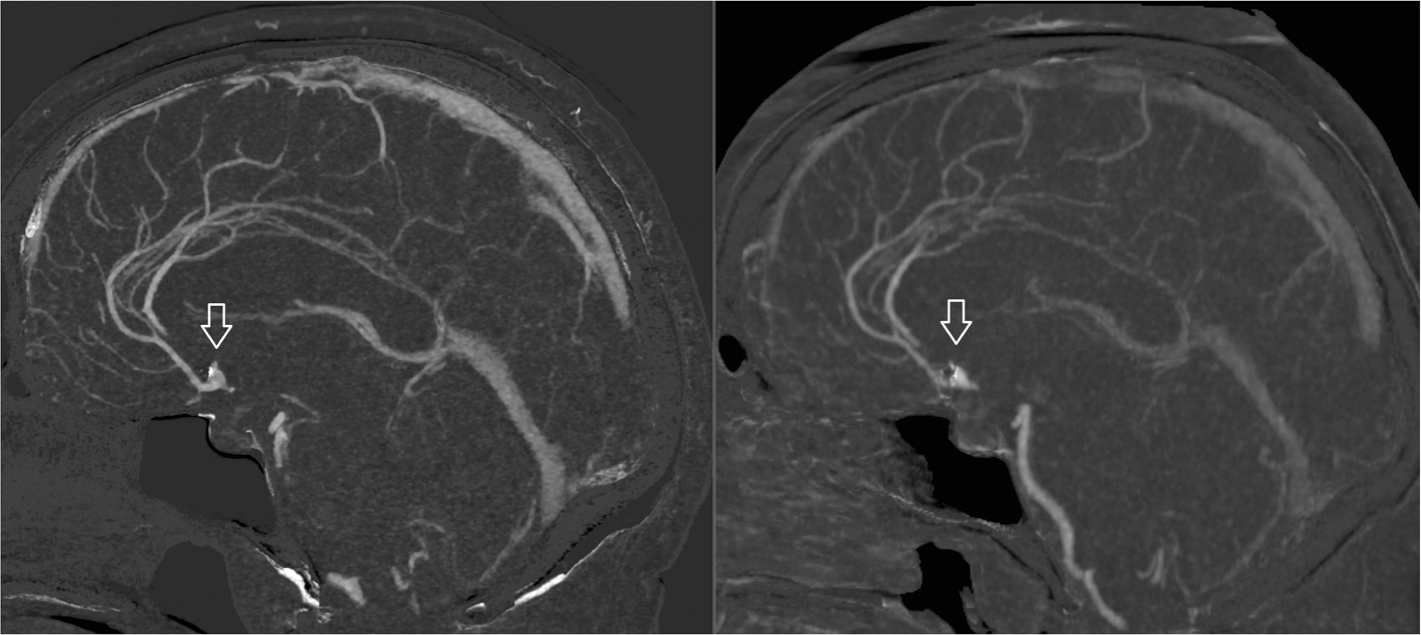
Sixty-nine-year-old female with surgical clip-treated anterior communicating aneurysm. Follow-up with subtraction CTA demonstrates a remnant of the aneurysm (arrows). Image quality of subtraction CTA is superior on the UHR system (left image) as compared to subtraction CTA on a conventional CT system (right image) due to increased spatial resolution (0.25 × 0.25 mm versus 0.5 × 0.5 mm)

**Fig. 3:**
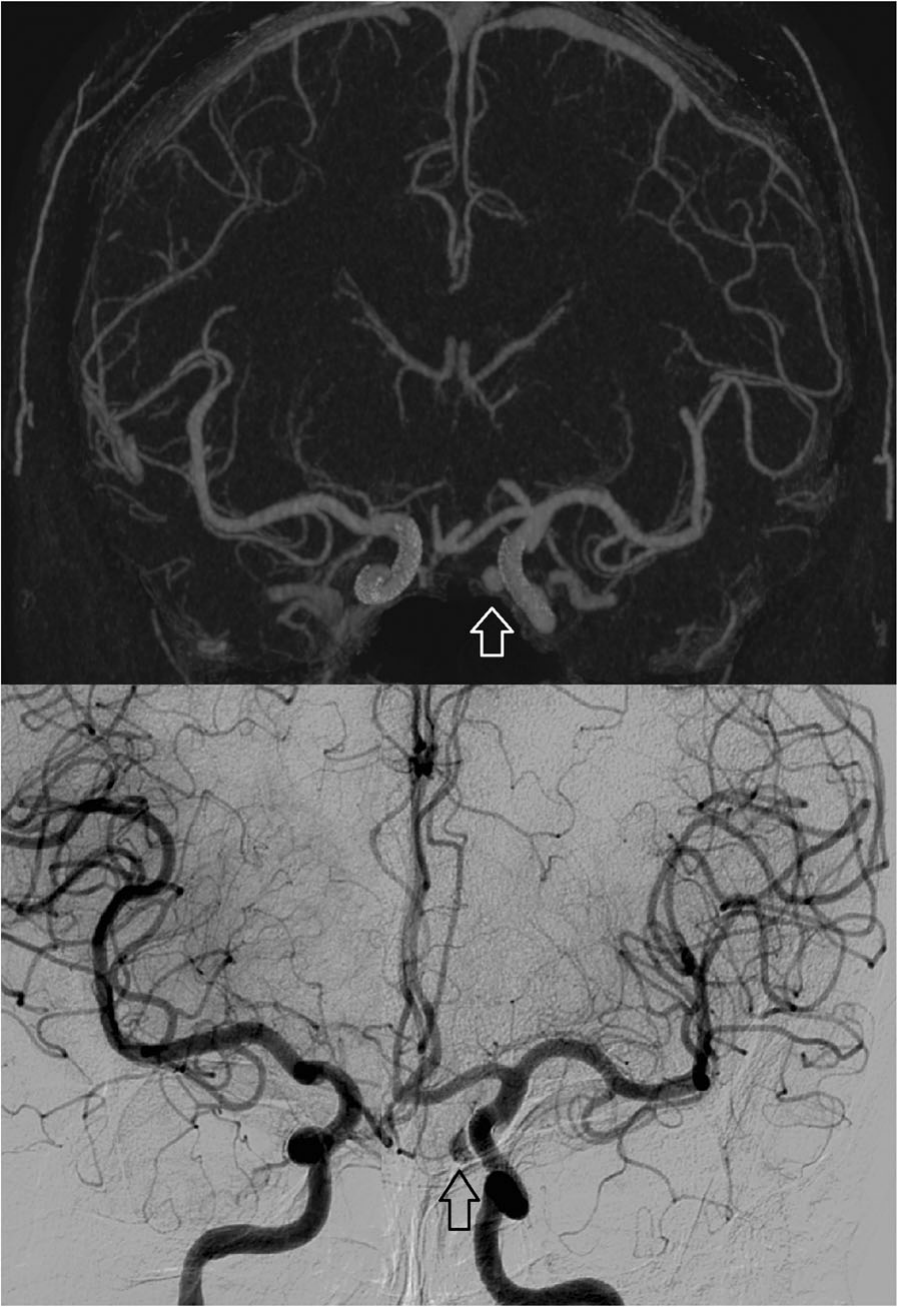
Fifty-seven-year-old female with flow diverter placement for treatment of internal carotid artery (ICA) aneurysms on both sides. At follow-up, occlusion of the right ICA aneurysm and residual contrast filling of the left ICA aneurysm (arrows) was seen with full consistency between UHR subtraction CTA (top) and conventional angiography (bottom)

**Fig. 4:**
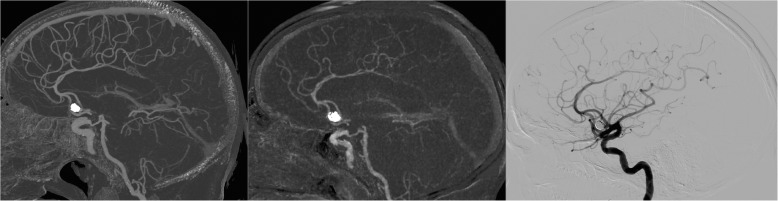
Sixty-seven-year-old male treated with coil occlusion of an anterior communicating artery aneurysm (same patient as in Fig. 1). Follow-up with UHR subtraction CTA (left image), subtraction CTA on a conventional CT system (middle image), and conventional angiography (right image) demonstrated complete occlusion of the aneurysm, with only limited artifacts from the coils

As UHRCT in combination with MBIR and MAR has only been recently introduced, our experience with cerebral sCTA using this system so far remains limited and no systematic evaluations are yet available. Previous investigations however have demonstrated the feasibility of UHRCT to improve the evaluation of smaller vascular structures as well as artifact reduction in other areas of the body [10–12]. Due to the increased spatial resolution and image quality, we now routinely apply UHR sCTA in the follow-up of treated intracranial aneurysms, examples of which are provided in Figs. 2, 3, and 4. In our initial experience, all cases have been of diagnostic image quality with the majority being rated as good to excellent. The corresponding median effective dose estimate of our protocol is around 2.4 mSv (mean DLP 1150, k-factor 0.0021), which is well in the range of standard reference levels and comparable to other CT scanners with lower spatial resolution [4, 13]. As compared to the standard resolution sCTA, smaller vascular structures are better delineated, which can be relevant for treatment planning. In addition, with application of MBIR and MAR, only limited artifacts resulting from the implanted materials are encountered, which allows a more confident evaluation of the treated aneurysm. Due to the increased spatial resolution, small untreated cerebral aneurysms are better depicted to evaluate the shape of the aneurysm, to identify branches originating from the aneurysm, and to appreciate the aneurysm’s relation with surrounding vessels, which is relevant for treatment planning. Of course, these experiences need to be verified in prospective cohort studies, where the added value of UHRCT over conventional spatial resolution needs to be evaluated. In selected patients, or when sCTA is inconclusive, DSA will probably still be of additional value because of its ability for selective vessel injection at high spatial and high temporal resolution, which enables dynamic evaluation of specific vessel structures and its small vessel branches.

## Conclusion

UHR sCTA is feasible and provides superior image quality in comparison to standard resolution sCTA, at similar radiation dose. It enables non-invasive vascular imaging with fine detail, approaching the resolution as seen during invasive DSA. With the application of optimized image filtering and MAR, it can be applied in the follow-up of treated cerebral aneurysms. However, the added value of UHR sCTA still needs to be evaluated in future prospective cohort studies.

## Abbreviations

CTA: CT angiography
DSA: Digital subtraction angiography
FIRST: Forward projected model-based Iterative Reconstruction SoluTion
MAR: Metal artifact reduction
MBIR: Model-based iterative reconstruction
MRA: MR angiography
sCTA: Subtraction CT angiography
SEMAR: Single-energy metal artifact reduction
UHR: Ultra-high resolution
UHRCT: Ultra-high-resolution CT
